# A randomized, controlled field study to assess the efficacy and safety of lotilaner flavored chewable tablets (Credelio™ CAT) in eliminating fleas in client-owned cats in the USA

**DOI:** 10.1186/s13071-021-04617-5

**Published:** 2021-03-01

**Authors:** Kimberly Chappell, Tandy Paarlberg, Wolfgang Seewald, Daniela Karadzovska, Steve Nanchen

**Affiliations:** 1 Elanco Animal Health, 2500 Innovation Way, Greenfield, IN 46140 USA; 2 Elanco Animal Health, Mattenstrasse 24A, 4058 Basel, Switzerland; 3 Elanco Australasia Pty Ltd, 245 Western Rd, Kemps Creek, NSW 2178 Australia

**Keywords:** Clinical field study, Credelio™ CAT, *Ctenocephalides felis*, Efficacy, FAD, Fipronil, Flea allergy dermatitis, Fleas, Frontline® plus, Lotilaner, Safety

## Abstract

**Background:**

Studies show that the novel isoxazoline, lotilaner (Credelio™ CAT; Elanco Animal Health), which is administered orally to cats, provides rapid and sustained flea kill for least 1 month following administration with a wide safety margin. A clinical trial was undertaken to confirm its efficacy, impact on flea allergy dermatitis (FAD) and safety under field conditions.

**Methods:**

A total of 343 cats were enrolled in the study at 11 veterinary clinics in the USA. Upon inclusion, cat households were randomized at a ratio of 2:1 to receive lotilaner tablets at the recommended dose (minimum 6 mg/kg) or a topical formulation containing fipronil + *S*-methoprene (Frontline® Plus for cats; Boehringer Ingelheim), administered per label. Owners were dispensed treatments for administration on days 0, 30 and 60; all household cats were administered the same treatment. Flea counts were made on primary cats (1 cat per household) on days 0 (pre-treatment), 30, 60 and 90. Flea allergy dermatitis was assessed on days 30, 60 and 90 for all cats with signs of FAD on day 0. Lotilaner-treated cats were also assessed for their acceptance of oral tablet administration by the pet owner, and safety was assessed for all cats in both groups.

**Results:**

Lotilaner efficacy was 98.3, 99.9 and 99.9% on days 30, 60 and 90, respectively, while the efficacy of fipronil + *S*-methoprene was 61.6, 75.4 and 84.7%, respectively (*P* < 0.0001, within both groups and all days). Flea counts were significantly lower in the lotilaner group than in the fipronil + *S*-methoprene group (*P* < 0.0001) on each assessment day. On day 90, 98.3% of lotilaner-treated cats and 28.8% of fipronil + *S*-methoprene-treated cats were free of fleas. Owners successfully administered 99.5% of tablets to their cats. Total FAD score was reduced significantly following treatment in both groups by day 30 (lotilaner: *P* < 0.0001; fipronil + *S*-methoprene: *P* = 0.0041) and continued to decrease following multiple treatments. Total FAD scores were also significantly lower in the lotilaner group than in the fipronil + *S*-methoprene group on day 90 (*P* = 0.0006 for FAD total score). Pruritus scores were significantly lower in the lotilaner group on all assessment days.

**Conclusion:**

A single lotilaner treatment, administered by the pet owner, was > 98% efficacious in reducing flea counts within 30 days. Three consecutive monthly lotilaner treatments resulted in nearly 100% reduction in flea infestation. In the evaluations of flea counts, number of cats free from fleas and pruritus FAD score, lotilaner was shown to be superior to fipronil + *S*-methoprene at all time points. Lotilaner was more efficacious than fipronil + *S*-methoprene and was associated with greater reduction in FAD signs. Lotilaner flavored tablets were well accepted by cats. Adverse reactions were mild and infrequent, confirming the safety of lotilaner tablets in client-owned cats.

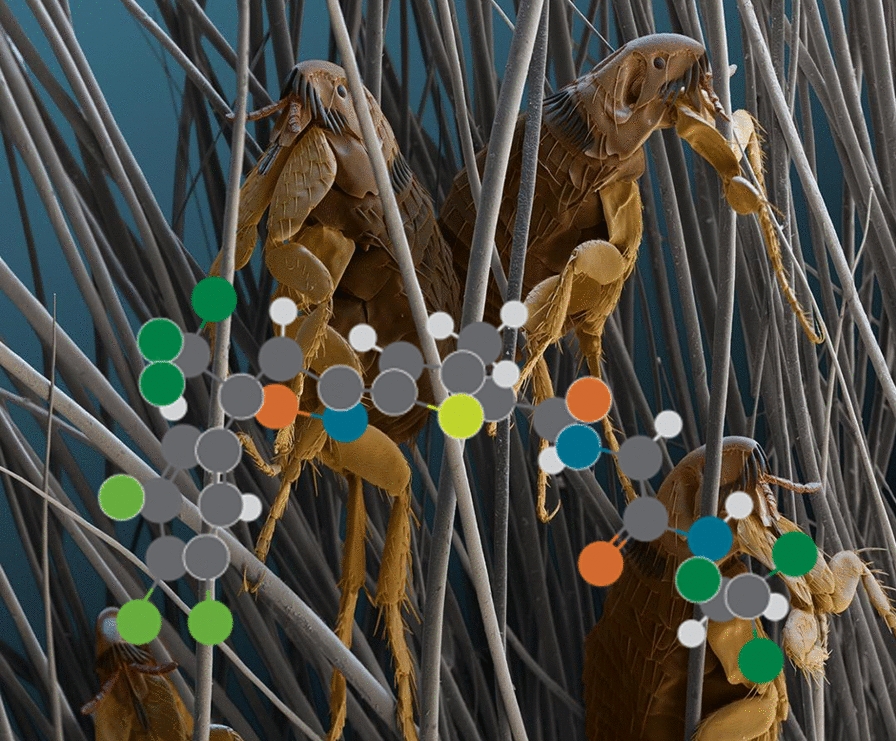

## Background

In much of the world, the cat flea (*Ctenocephalides felis*) is the most commonly encountered ectoparasite of cats and dogs [1]. In addition to being a nuisance and irritant, uncontrolled flea populations can cause miliary dermatitis, self-inflicted trauma, alopecia due to pruritus and anemia, as well as more severe dermatological conditions including flea allergy dermatitis (FAD) [1, 2]. The injection of antigens present in flea saliva as the parasite feeds can cause a hypersensitive state in some cats, and once sensitization occurs, dermatologic lesions can recrudesce following reinfestation [2]. Fleas are known vectors of zoonotic organisms, such as *Rickettsia* spp. and *Bartonella* spp., and serve as intermediate hosts of the tapeworm *Dipylidium caninum*, adult stages of which have been reported to develop in children upon ingestion of infected fleas from infested animals [1, 3]. In the absence of control measures, flea infestations can contaminate household environments, leading to potential flea bites of human occupants [4].

Female fleas begin laying eggs within 24 to 36 h after finding a host, and they can lay as many as 50 eggs per day. Cat fleas can survive more than 100 days on a host, with continued daily egg production [5]. Flea eggs fall from the animal host, feeding the cycle of immature flea life stages in the host’s environment and sustaining the infestation. Environmental pesticides employed to eliminate flea populations from households may not be effective and their use exposes household inhabitants to pesticide residues [6]. An appropriate control strategy for fleas and flea infestations therefore includes the use of products that eliminate existing fleas on pets and protect against post-treatment challenges from flea life stages present in a contaminated environment.

Lotilaner, a novel isoxazoline, provides veterinarians and pet owners with an effective, fast-acting and long-lasting measure to control feline flea infestations. In a margin of safety study, lotilaner was well tolerated by kittens 8 weeks old at study start, at doses of up to 130 mg/kg (corresponding to 5-fold the maximum recommended dose), administered monthly over 8 months [7]. Results from laboratory efficacy studies demonstrate that lotilaner starts killing fleas on cats within 6 to 8 h after administration [8, 9], and an efficacy of 100% against fleas was demonstrated through to the end of the study (35 days after treatment initiation), indicating that monthly use of lotilaner will effectively eliminate flea life-cycle stages from the cat’s environment [8].

The field study described here was designed to confirm the results of prior laboratory efficacy and safety studies. The primary study objective was to evaluate the efficacy and safety of lotilaner flavored chewable tablets administered orally by cat owners at the recommended dose (minimum 6 mg/kg) for the treatment and prevention of flea infestations. Secondary study objectives were to evaluate the presence and persistence of clinical signs associated with FAD (pruritus, erythema, scaling, papules, alopecia and dermatitis/pyodermatitis) and to evaluate the oral administration acceptance of the tablet formulation.

## Methods

This was a randomized, blinded, positive controlled field study in cats enrolled by participating veterinary practices across the USA. The protocol was prepared in compliance with the World Association for the Advancement of Veterinary Parasitology (WAAVP) guidelines for evaluating the efficacy of parasiticides for the treatment, prevention and control of flea and tick infestation on dogs and cats [10]. The study was conducted and documented in accordance with the U.S. Food and Drug Administration–Center for Veterinary Medicine (FDA-CVM) Guidance for Industry 85, International Co-operation on Harmonisation of Technical Requirements for Registration of Veterinary Products (VICH) GL9, *Good Clinical Practice* (May 2001) [11].

### Animals and households

Households enrolled in the study were required to have at least one and no more than three cats. All household cats were to be at least 8 weeks of age, weigh at least 2 pounds and be clinically in good health or have minor ailments that would not be expected to interfere with the study. Cats with pre-existing chronic diseases (i.e. diabetes, hyperthyroidism, osteoarthritis) were eligible for inclusion if these conditions were considered to be stable or controlled. At least one cat in each qualifying household was required to have a minimum of five fleas.

Households with the following situations were excluded from enrollment: if they contained cats that were intended for breeding or were pregnant or lactating; if there had been any environmental flea treatment within 30 days prior to study participation; or if they contained cats treated with products likely to interfere with the conduct or interpretation of study results (e.g. treatment with another ectoparasiticide preparation). Minimum withdrawal times which could interfere with study results were established for ectoparasiticide treatments, and these corresponded to the efficacy duration given on the product label. If the product used was not clearly identified, a minimum withdrawal time of 4 weeks was followed. A minimum withdrawal time of 2 weeks prior to study participation was applied for use of an ectoparasiticide collar. Bathing/shampooing of study cats was not permitted within 48 h prior to the start of the study treatment in order to avoid confounding factors which could potentially impact the day 0 flea counts.

Other than for that one pretreatment restriction, or use of any product active against fleas and/or ticks, there were no restrictions on wetting or bathing and no restrictions on the presence of non-feline household pets. Cats were permitted to be kept indoors or outdoors. Dogs in study households were required to receive treatment for the duration of the study with a monthly flea adulticide product that was commercially available and dispensed by the clinic at the time of study initiation.

The primary cat in each household was the experimental unit. The primary cat was chosen by random drawing if more than one cat in the household met all inclusion criteria, including a minumum of five fleas. All household cats dosed with the study treatment were included in the safety analyses. Participating study cats were fed, housed and managed by their owners, and pet owners were requested to maintain study cats on the same diet throughout the study. Cat owners administered study treatments. Participating veterinary clinics followed standard veterinary procedures.

Permitted reasons for study withdrawal included: discretion of the investigator, owner withdrawal of consent or an adverse event requiring cessation of study treatment or observations. Other withdrawal reasons included: use of a protocol-restricted concomitant treatment, lack of study treatment efficacy, loss to follow-up and protocol deviations which may have compromised study integrity.

### Enrollment

Each clinic performed a thorough physical examination, including body weight, of all study cats and determined a body condition score. An assessment of FAD signs was performed for any cat with at least one flea. Blood samples were collected from cats for serum chemistry and hematology testing to serve as a baseline assessment of health. All cats were combed for flea and tick counts. The household was enrolled in the study once all cats were determined to be eligible, and at least one cat had a minimum of five fleas. Within each clinic, primary cats were then blocked into threes on order of household enrollment. Primary cats were then randomly allocated to a treatment group using a 2:1 ratio of lotilaner (Credelio™ CAT; Elanco Animal Health, Greenfield, IN, USA) to fipronil + *S*-methoprene (Frontline® Plus; Boehringer Ingelheim Animal Health USA Inc., Duluth, GA, USA). Other household cats were designated as supplementary cats and received the same treatment as the household primary cat. Flea and tick counts were not performed at subsequent visits for supplementary cats. FAD assessments were performed at subsequent visits on all cats (both primary and supplementary) if their enrollment visit flea count was at least one flea and if FAD signs were present at enrollment. The target enrollment was 100 primary cats in the lotilaner treatment group and 50 primary cats in the fipronil + *S*-methoprene treatment group.

### Treatments

Treatments dispensed to owners for administration at home were: (i) Lotilaner (Credelio™ CAT) available to each clinic for dispensing in two tablet strengths, namely 12 and 48 mg lotilaner, to be administered on the basis of each cat’s body weight at the proposed label dose of 6–25 mg/kg; (ii) fipronil + *S*-methoprene (Frontline® Plus), available to each clinic for dispensing as individual dose applicators for topical administration (fipronil 9.8% and *S*-methoprene 11.8% per 0.5-ml applicator), to be administered per label.

Examining veterinarians who conducted flea/tick counts, physical examinations and FAD assessments and determined body condition scores were blinded to treatment asignments. Designated study personnel at each site dispensed study treatments to owners and were responsible for treatment group allocation, training pet owners on study treatment administration and drug accountability. Treatment records were maintained separately from those of the examining veterinarian’s records, and designated treatment personnel did not disclose treatment-related information to the examining veterinarian (and/or trained designees). At each visit, the designated treatment personnel dispensed the appropriate treatment for each household cat to the pet owner. Owners were instructed to administer study treatments once on each of days 0, 30 (± 2) and 60 (± 2) and to feed their cats within approximately 30 min prior to lotilaner treatment.

For animals on lotilaner treatment, each cat’s owner was instructed to initially offer the tablet by hand, on the floor or in an empty bowl. If the cat did not accept and consume the tablet, the owner was instructed to offer the tablet in a small amount of food or in a treat. If the cat did not voluntarily accept the tablet, the owner was instructed to administer the tablet directly into the cat’s mouth, towards the back of the tongue, and then to encourage the cat to swallow the tablet. If the cat vomited the tablet within 30 min of administration, the owner was instructed to contact the treatment dispenser at the clinic in order to obtain a replacement.

Concomitant treatments were permitted, provided they were not expected to interfere with study objectives. Some concomitant treatments, such as corticosteroids, antihistamines and antibiotics administered for FAD signs, required exclusion of the FAD assessment data. Products such as vaccinations, heartworm preventives, intestinal parasiticides, or nutritional supplements were considered acceptable.

### Flea counting and assessment of flea allergy dermatitis

Each primary cat in the household was subjected to flea comb counts on study days 0, 30, 60 and 90. Flea comb counts were performed on day 0 only for all supplementary cats. To perform the counts, study personnel used an extra fine-toothed flea comb and combed the entire body of each cat for a minimum of 10 min. At the initial visit, study personnel stopped counting if fewer than five fleas were counted within the initial 5 min of combing. Heavy infestations consisting of > 250 fleas were recorded as > 250. For these cats, the value of 251 was used for analysis. All fleas removed during the comb count procedure were disposed of and were not returned to any cats.

On day 0, the examining veterinarian performed an FAD assessment for each study cat with a flea count of at least one. Subsequent FAD assessments were performed on days 30, 60 and 90 for any cats demonstrating FAD signs on day 0. Each sign of FAD (pruritus, papules, erythema, alopecia, scaling, dermatitis/pyodermatitis) was assessed and classified as absent, mild, moderate or severe, corresponding to a scoring of 0 (absent) to 3 (severe). Wherever possible, the same examining veterinarian performed each follow-up FAD assessment for each cat. FAD assessments were not performed on days 30, 60 and 90 for any cats with no fleas on day 0, but any subsequent appearance of FAD signs was recorded as an adverse event.

### Assessments and statistics

The efficacy of each treatment in the control of flea infestation was assessed by comparing baseline flea counts on day 0 (enrollment visit) with those at 30, 60 and 90 days later. Efficacy was determined* via* the percentage reduction in live adult flea counts from pre- to post-dosing within each treatment group. The calculation used for percentage efficacy at each counting time was: percentage efficacy = ([MB–MA]/MB) × 100, where MB is the mean flea count prior to dosing (day 0) and MA is the mean flea count post-dosing (days 30, 60 and 90, respectively).

Geometric means were used in the calculations for efficacy determination, and calculations were also performed using arithmetic means. Calculation of geometric means involved taking the logarithm of the flea count of each cat. For any flea counts equal to zero, 1 was added to the count for every animal in the group and then subtracted from the resultant mean prior to calculating percentage efficacy. For each treatment group, the log-transformed flea (count + 1) data were analyzed from pre- to post-dosing with analysis of variance (ANOVA) to determine if a statistically significant flea count reduction from baseline occurred at each time point. The model included a fixed effect, Paired, which was defined as an indicator variable (0, 1; to represent pre- and post-dosing counts), and the random effect of Site. Separate models were fitted for each of days 30, 60 and 90 compared to day 0.

A study treatment was considered to be efficacious at any time point if the following criteria were met: (i) cats were adequately infested with fleas prior to first dosing (≥ 5 fleas); (ii) the calculated efficacy at the time point was ≥ 90%, based on geometric means; (iii) there was a statistically significant decrease at a two-sided 0.05 level of significance (*P* < 0.05) in pre- to post-dosing flea counts at the time point.

Flea counts were compared between the treatment groups, separately for each of days 30, 60 and 90, using ANOVA. Likewise, the number of cats free of fleas was compared using Fisher’s exact test.

As a secondary efficacy variable, total FAD score (for all cats receiving post-enrollment FAD assessments) was calculated at each time point as the sum of the clinical sign scores (pruritus, erythema, scaling, papules, alopecia, dermatitis/pyodermatitis scores) and evaluated over time by an ANOVA. Differences between treatment groups for each of days 30, 60 and 90 were determined, as well as within a treatment group over time. An additional secondary endpoint was the acceptance of the lotilaner tablets. Tablet acceptance was assessed by the cat owner at each of the dosing episodes on days 0, 30, and 60.

Safety was assessed through the evaluation of adverse events reported by the pet owner and the evaluating veterinarian. Body weight was statistically evaluated over time and within each treatment group. Clinical pathology parameters (hematology and clinical chemistry variables) were evaluated descriptively relative to normal ranges provided by the laboratory and in the context of pre- and post-dosing values.

All statistical analyses were performed using the SAS® software package (SAS Institute, Cary, NC, USA).

## Results

From June to November 2015, 139 primary cats were enrolled in the lotilaner group (230 cats in total, including supplementary household cats) and 69 primary cats were enrolled in the fipronil + *S*-methoprene group (113 cats in total) at 11 veterinary clinics across the USA (2 clinics in each of Florida and Missouri, and 1 clinic in each of Arkansas, California, Louisiana, Michigan, Oregon, South Carolina and Texas). The safety population was defined as enrolled cats which received at least one dose of the study treatment and included 228 cats in the lotilaner group (dosing could not be confirmed for 2 enrolled cats, and these were excluded from the safety population) and 113 cats in the fipronil + *S*-methoprene groups. The mean age of cats in the safety population of both groups was approximately 5 years (range 1.9 months to 20 years) (Table 1). There was a similar distribution of age groupings per treatment group, and over 75% of cats in each group were older than 12 months. The minimum ages were 1.9 months in the lotilaner group and 2.3 months in the fipronil + *S*-methoprene group, and minimum weights were 0.9 and 1.1 kg, respectively. The sex and neuter status of enrolled cats was similar in both groups, as was the distribution of single and multiple cat households. Domestic short-haired cats were the most common breed enrolled (81.1% of cats in the lotilaner group and 77.0% of cats in the fipronil + *S*-methoprene group). Domestic long-haired cats represented 8.8 and 13.3% of the lotilaner group and fipronil + *S*-methoprene group, respectively. Other breeds enrolled in the study, but represented at lower frequencies, were Siamese, Persian, Himalayan, Bengal, Sphinx, Ragdoll, Russian Blue and Egyptian Mau.

**Table 1:** Demographics of enrolled cats

Demographics	Lotilaner treatment group (*n* = 228)	Fipronil + *S*-methoprene treatment group (*n* = 113)
Age (months)		
Mean	56.6	58.1
SD	52.4	56.4
Median	36	36
Range	1.9–180.0	2.3–240.0
Weight (kg)		
Mean	4.3	4.2
SD	1.7	1.5
Median	4.3	4.2
Range	0.9–10.1	1.1–9.0
Female, intact (*n*)	29 (12.7%)	15 (13.3%)
Sex (*n*)		
Female, spayed	92 (40.4%)	44 (38.9%)
Male, intact	18 (7.9%)	12 (10.6%)
Male, neutered	89 (39.0%)	42 (37.2%)
Domestic short hair	185 (81.1%)	87 (77.0%)
Breed (*n*)		
Domestic long hair	20 (8.8%)	15 (13.3%)
Other	23 (10.1%)	11 (9.7%)
At least 12 months (*n*)	172 (75.5%)	94 (83.2%)
Age category (*n*)		
< 12 months and at least 4 months	32 (14.0%)	12 (10.6%)
< 4 months	24 (10.5%)	7 (6.2%)

Numbers are based on study cats in the safety population, defined as enrolled cats which received at least one study treatment

SD, Standard deviation

For assessments of efficacy, data were used from 126 and 62 primary cats treated with lotilaner and fipronil + *S*-methoprene, respectively. Reasons for exclusion of cats and data in the efficacy analyses included loss of follow-up, cat not fed prior to lotilaner dose, forbidden concomitant medication, occurrence of an adverse event that required stopping the observations, withdrawal of owner’s consent and death (2 primary cats died, discussed below).

**Fig. 1:**
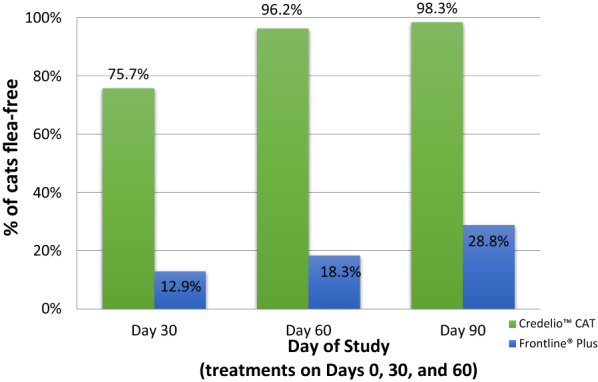
Percentage of primary cats in each group that were free of fleas at each post-treatment assessment on days 30, 60 and 90. Difference between groups was significant at each time point at *P* < 0.0001

**Fig. 2:**
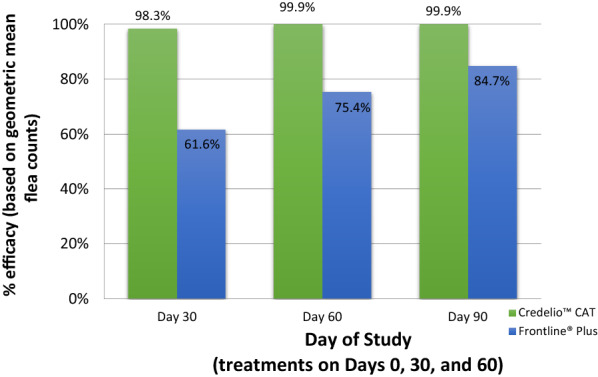
Percentage efficacy based on geometric mean flea counts at post-treatment assessment on days 30, 60 and 90. Difference between groups was significant at each time point at *P* < 0.0001

Both groups showed statistically significant (*P* < 0.0001; Table 2; Fig. 2) reductions in mean flea counts from baseline (pre-treatment, day 0 visit) at every post-treatment assessment up to the end of the study. At day 90, 98.3% of lotilaner-treated cats and 28.8% of fipronil + *S*-methoprene-treated cats were free of fleas (Table 3; Fig. 1). This difference in proportions of cats free of fleas between the two treatment groups was statistically significant (*P* < 0.0001, *Χ*_1_^2^ = 63.8) (Table 3; Fig. 1). The statistical comparison of the treatment groups also showed that there were significantly fewer fleas on cats in the lotilaner group than in the fipronil + *S*-methoprene group on days 30 (*t*_(8)_  = 3.49, *P* = 0.0081), 60 (*t*_(8)_  = 5.02, *P* = 0.0010) and 90 (*t*_(8)_  = 8.01, *P* < 0.0001) (Table 4); flea counts on day 0 were not statistically different (*t*_(8)_ = 0.45, *P* = 0.66). Efficacy, based on geometric mean flea counts, for the lotilaner group was 98.3% on day 30 and 99.9% on days 60 and 90; for the fipronil + *S*-methoprene group, efficacyt was < 90% for all post-treatment time points (Table 2; Fig. 2).

**Table 2:** Flea count data for each treatment group and statistical analysis of flea count reduction from baseline for each treatment group

Flea count data	Lotilaner treatment group	Fipronil and* S*-methoprene treatment group
Day 0 (pre-treatment)		
Arithmetic mean ± SD	46.3 ± 61.0	45.3 ± 59.5
Range^a^	5–251	5–251
Geometric mean	24.6	25.8
Day 30		
Arithmetic mean ± SD	1.3 ± 5.1	27.3 ± 52.0
Range	0–48	0–251
Geometric mean	0.4	9.9
% Efficacy^b^	98.3	61.6
Statistical analysis	*t*_(114)_ = 29.05, *P* < 0.0001	*t*_(61)_ = 6.86, *P* < 0.0001
Day 60		
Arithmetic mean ± SD	0.0 ± 0.2	17.6 ± 36.0
Range	0–1	0–184
Geometric mean	0.0	6.3
% Efficacy^b^	99.9	75.4
Statistical analysis	*t*_(203)_ = 32.65, *P* < 0.0001	*t*_(59)_ = 8.86, *P* < 0.0001
Day 90		
Arithmetic mean ± SD	0.1 ± 0.6	9.0 ± 12.0
Range	0–6	0–59
Geometric mean	0.0	4.0
% Efficacy^b^	99.9	84.7
Statistical analysis	*t*_(221)_ = 32.94, *P* < 0.0001	*t*_(51)_ = 9.63, *P* < 0.0001

^a^Range: any count > 250 fleas was assigned the value 251

^b^% Efficacy: based on geometric means

**Table 3:** Number and percentage of cats with zero flea counts at each time point

Day	Lotilaner			Fipronil and* S*-methoprene treatment group			Fisher’s exact test for comparison
	*n*	No. cats with zero fleas	Percentage	*n*	No. cats with zero fleas	Percentage	
30	115	87	75.7	62	8	12.9	*Χ*_1_^2^ = 63.8, *P* < 0.0001
60	106	102	96.2	60	11	18.3	*Χ*_1_^2^ = 107.0, *P* < 0.0001
90	115	113	98.3	52	15	28.8	*Χ*_1_^2^ = 96.4, *P* < 0.0001

**Table 4:** Analysis of variance between-group comparison of flea counts (log-transformed)

Response	Day	Lotilaner treatment group	Fipronil and * S*-methoprene treatment group	*df*	*t*	*P*
Flea count	30	1.274	26.53	8	− 3.49	0.0081*
	60	0.038	17.55	8	− 5.02	0.0010*
	90	0.061	9.04	8	− 8.01	< 0 .0001*

*Significantly fewer fleas on cats in the lotilaner group than in the fipronil + *S*-methoprene group

Tablet acceptability, as administered by pet owners, was documented for all lotilaner-treated cats. Lotilaner tablets were voluntarily accepted by 46.9% of cats when offered by hand, in an empty food bowl or with food. The tablets were administered directly into the mouth for 52.6% of cats. In the study, 99.5% of tablets were successfully administered (Table 5). No cats were observed to have vomited within 30 min of treatment. Redosing was reported once each for two cats in the study (Table 5) due to pet owner difficulty in dosing. These cats were then successfully dosed with the assistance of study site personnel.

**Table 5:** Summary of lotilaner tablet acceptance

Study visit	Number of cats^a^	Tablet(s) accepted voluntarily (%)^b,c^	Tablet(s) Accepted Placed in Mouth (%)^b,d^	Tablet(s) successfully dosed by pet owner (%)^b^	Tablet(s) could not be administered by pet owner (%)^b,e^
Day 0	225	48.0	52.0	100	0.0
Day 30	213	49.3	50.2	99.5	0.5
Day 60	210	43.3	55.7	99	1.0
All doses	648	46.9	52.6	99.5	0.5

^a^Only cats with data on how the tablet was accepted were included in the number of cats available

^b^The denominator for all percentages calculated is the number of cats in the respective row of the table

^c^Voluntary acceptance refers to tablets consumed unaided (by the hand, on the floor or empty food bowl) or with the addition of food

^d^Placed in mouth refers to a cat that consumed the tablet(s) after tablet was placed at the back of the mouth

^e^Refers to doses which could not be administered by the pet owner. Two cats were successfully redosed by clinic study staff

There were 58 cats in the lotilaner group and 27 cats in the fipronil + *S*-methoprene group which had FAD clinical signs monitored and were eligible for analysis throughout the study (i.e. FAD population). The arithmetic mean total FAD scores at baseline were 4.2 in the lotilaner group and 4.4 in the fipronil +* S*-methoprene group. Analysis of the FAD total score indicated that the total score was reduced significantly following treatment in both groups by day 30 (lotilaner group: *P* < 0.0001; fipronil + *S*-methoprene group: *P* = 0.0041) and continued to decrease following multiple treatments, falling to 1.0, 0.3, 0.1 on days 30, 60 and 90 (lotilaner group) and to 3.0, 2.5, 2.0 (fipronil + *S*-methoprene group), respectively. This reduction was statistically significant for both groups on all days. The FAD total score was also significantly lower in the lotilaner group than in the fipronil + *S*-methoprene group on day 90 (*t*_(7)_ = 5.83, *P* = 0.0006). FAD scores for pruritus were significantly lower in the lotilaner group than in the fipronil + *S*-methoprene group on all post-treatment days (*P* = 0.0420 for day 30; *P *= 0.0397 for day 60; *P* = 0.0033 for day 90).

Adverse reactions that occurred at an incidence of ≥ 1% are presented in Table 6. Weight loss was reported with similar frequency in both groups (lotinaner group: 2.2%; fipronil + *S*-methoprene group: 1.8%). Tachypnea was reported in three cats (1.3%) from the same household in the lotilaner group. This adverse reaction for all three cats was observed on day 0 and was not observed after dosing on days 30 or 60. Vomiting was reported in 1.3% of the lotilaner group and 0.9% of the fipronil + S-methoprene group.

**Table 6:** Cats experiencing the most common adverse reactions reported during the study

Adverse reaction	Lotilaner treatment group (*n* = 228)	Fipronil + S-methoprene treatment group (*n* = 113)
Weight loss	5 (2.2%)	2 (1.8%)
Tachypnea	3 (1.3%)	0 (0.0%)
Vomiting	3 (1.3%)	1 (0.9%)

Values in table are presented as the number of cas with the adverse reaction, with the percentage in parentheses

Two cats died during the study, one from each group. The cause of death for each cat was motor vehicle trauma and was not considered to be an adverse reaction to the study treatment. Two cats, one from each group, were removed from the study prematurely due to adverse events. The cat in the lotilaner group was removed from the study and diagnosed with disseminated lymphosarcoma. The cat in the fipronil + *S*-methoprene group suffered a broken jaw due to motor vehicle trauma and was removed from the study. Both of these events were not attributed to treatment.

Analyses of of hematology and clinical chemistry identified individual and isolated departures from normal reference ranges, but did not reflect patterns of clinical significance in terms of safety of the study treatments.

A range of concomitant medications were administered to study cats, including some not allowed by the protocol, ranging from antibacterial medications, anti-inflammatory medications (including corticosteroid-containing products), endoparasiticide medications, sedatives and vaccines. Data from cats receiving concomitant medications for treatment of FAD signs were excluded from the FAD analyses. No other ectoparasiticide products (other than study treatments) were used during the study. All concomitant treatments were well tolerated.

## Discussion

The diverse group of cats enrolled in the lotilaner group and their broad geographic spread across the different regions of the USA provide a good representation of the real-world conditions regarding the use of an antiparasitic product. The study spanned the seasons of summer, fall and early winter in the temperate climate of the USA and, therefore, study households and cats were exposed to seasonal factors (such as warm temperatures and humidity) which are hospitable to flea infestations and increase the potential for the development of signs of FAD [2]. The results of this study align with results of laboratory studies demonstrating lotilaner to be a safe and efficacious flea control treatment for cats [7–9]. Lotilaner was shown to be highly efficacious from the first treatment onward. Efficacy, based on geometric mean flea counts, for the lotilaner group was 98.3% on day 30 and 99.9% on days 60 and 90, compared to 61.6, 75.4, and 84.7% for the fipronil + *S*-methoprene group, respectively, and the percentage of flea free cats by day 90 was 98.3% for the lotilaner group and 28.8% for the fipronil + *S*-methoprene group (Table 3; Fig. 1). With respect to flea counts, the number of cats free from fleas and total FAD score, lotilaner was shown to be superior to fipronil + *S*-methoprene.

The FAD signs scoring system used in this study (scoring lesions as mild, moderate and severe) is consistent with that described for other field studies assessing the efficacy of isoxazoline flea control products [12–14]. Limitations of this scoring methodology include that it has not been validated and it is subjective, whereby grading may vary between clinicians. Nonetheless, as in other reported studies, the progressive and marked improvement in each clinical sign of FAD in treated cats can be attributed to two factors that are related to lotilaner’s rapid onset and sustained residual flea speed of kill [8, 9]. The first is the reduction of antigenic challenge* via* the rapid kill of newly infesting fleas. The second factor is due to the killing of newly infesting fleas on the cat before egg-laying can begin, which promotes progressive depletion of household flea biomass and subsequent elimination.

The results in this study align with those reported from a European field study in which lotilaner efficacy in 121 primary cats was 97.2 and 98.1% on days 14 and 28, compared to 48.3 and 46.4% for a topical fipronil + *S*-methoprene monthly product [12]. While Credelio™ CAT (lotilaner flavored chewable tablet) is the first orally administered isoxazoline product for cats in the USA, two feline topical isoxazolines have been approved by the FDA [13, 14]. A study in the USA on a topical isoxazoline formulation for administration every 12 weeks compared the results of 195 topical fluralaner-treated cats and 98 cats treated with a monthly topical fipronil + *S*-methoprene formulation. At 4, 8, and 12 weeks post-administration, the efficacy of fluralaner was > 99%, and the percentage of flea-free cats was 80.7, 88.7 and 80.0%, respectively [13]. A study in the USA on a topical isoxazoline combination formulation (selamectin + sarolaner) compared the results of 171 cats treated with selamectin + sarolaner and 82 cats treated with a topical imidacloprid + moxidectin formulation for three consecutive monthly doses. Efficacy of the selamectin + sarolaner formulation was > 97% at 30, 60 and 90 days post-treatment initiation [14]. Overall, these findings indicate that the performance of orally administered lotilaner, under field conditions, is consistent with the performance reported for topical isoxazoline formulations.

An important attribute of a product to control fleas in cats is the ease of administration of the product for the cat owner. Overall, 46.9% of the tablets were accepted voluntarily (e.g. offered from the hand, floor, empty food bowl or with the addition of food). Combining voluntary acceptance with doses which were delivered to cats by being placed in the mouth (52.6%) gave an overall tablet acceptance of 99.5% when administered by the cat owners. Only 0.5% of the doses could not be administered by pet owners, but these cats were subsequently and successfully redosed. No cats were withdrawn because of inability to administer treatment. The study results therefore confirm the acceptance of the lotilaner flavored tablet formulation for cats.

Adverse reactions were mild and infrequent, confirming the safety of lotilaner in client-owned cats.

## Conclusion

The results of this study, utizing client-owned cats across the USA, demonstrate the safety and efficacy of lotilaner tablets under a wide range of real-world conditions. Lotilaner flavored chewable tablets were easily administered by owners and widely accepted by cats. A single lotilaner treatment, administered by the pet owner, was 98.3% efficacious in reducing mean flea counts within 30 days, the time of the first post-treatment assessment. Three consecutive monthly lotilaner treatments resulted in a >99.9% reduction in flea infestations. In comparison, treatment with fipronil + *S*-methoprene was < 90% efficacious throughout the study. Treatment with lotilaner resulted in a marked reduction in, or elimination of, signs of flea allergy dermatitis throughout the 3-month study. Lotilaner was more efficacious for the treatment and control of flea infestations than fipronil + *S*-methoprene and was associated with greater reduction in signs of FAD. Adverse reactions were mild and infrequent, confirming the safety of lotilaner in client-owned cats. The study therefore demonstrates that lotilaner flavored chewable tablets (Credelio™ CAT) are widely accepted and easy to administer, and that the safety and efficacy of lotilaner are maintained regardless of geography and season.

## Data Availability

Due to commercial confidentiality of the research, data not included in the manuscript can only be made available to *bona fide* researchers subject to a non-disclosure agreement.

## Abbreviations

FAD: Flea allergy dermatitis
FDA-CVM: United States Food and Drug Administration–Center for Veterinary Medicine
